# Impaired emotional awareness is associated with childhood maltreatment exposure and positive symptoms in schizophrenia

**DOI:** 10.3389/fpsyt.2023.1325617

**Published:** 2024-01-11

**Authors:** Kendall Beals, Lénie J. Torregrossa, Ryan Smith, Richard David Lane, Julia M. Sheffield

**Affiliations:** ^1^Sheffield Lab, Department of Psychiatry and Behavioral Sciences, Vanderbilt University Medical Center, Nashville, TN, United States; ^2^Social Cognition and Recovery in Schizophrenia Lab, Department of Psychology, The University of Southern Mississippi, Hattiesburg, MS, United States; ^3^Laureate Institute for Brain Research, Tulsa, OK, United States; ^4^Department of Psychiatry, University of Arizona, Tucson, AZ, United States

**Keywords:** emotional awareness, schizophrenia, childhood maltreatment, positive symptoms, indirect effect

## Abstract

**Objectives:**

Evidence suggests that emotional awareness—the ability to identify and label emotions—may be impaired in schizophrenia and related to positive symptom severity. Exposure to childhood maltreatment is a risk factor for both low emotional awareness and positive symptoms.

**Methods:**

The current investigation examines associations between a performance-based measure of emotional awareness, positive symptom severity, and childhood maltreatment exposure in 44 individuals with a schizophrenia-spectrum disorder and 48 healthy comparison participants using the electronic Levels of Emotional Awareness Scale (eLEAS), Positive and Negative Syndrome Scale (PANSS) and Childhood Trauma Questionnaire (CTQ).

**Results:**

Patients demonstrated significant deficits in emotional awareness overall, which was true for both self and others. In patients, lower emotional awareness was significantly associated with more severe positive symptoms. Emotional awareness was significantly impaired in patients with schizophrenia with self-reported maltreatment exposure, relative to other groups. Severity of maltreatment was not significantly associated with emotional awareness or positive symptoms when looking continuously, and there was no significant indirect effect.

**Conclusion:**

These data suggest that emotional awareness impairments observed in schizophrenia may be exacerbated by exposure to childhood maltreatment, possibly putting individuals at greater risk for experiencing positive symptoms of psychosis.

## 1 Introduction

Schizophrenia is a serious mental health disorder associated with a heterogeneous presentation of symptoms, in addition to early markers of dysfunction and potential for long-term disability. Alterations in emotional processes are commonly observed in individuals with schizophrenia and have been noticed as a core aspect of the disorder from the time of Kraepelin (1). A greater understanding of why altered emotional processing may manifest in schizophrenia and its relation to symptom severity is critical for identifying clinical targets.

Prior work has suggested that emotional awareness is one aspect of emotional processing that is impaired in schizophrenia (2–6) as well as individuals high in schizotypy (7). Emotional awareness is the ability to identify, interpret and describe emotions (8, 9) reflecting a subjective understanding of emotional states. Emotional awareness is a critical precursor to emotion regulation, as recognition of emotional states can support decision-making and management of emotional experiences (10–13). Accordingly, low emotional awareness has been linked to a range of mental health difficulties, including schizophrenia (14–16).

Deficits in emotional awareness in schizophrenia has been observed when measured using self-reported emotional awareness (6, 8, 9, 15) and performance-based measures such as the Levels of Emotional Awareness Scale (LEAS) (15, 16). For instance, individuals with schizophrenia provide less appropriate descriptions of emotions in a given context (17). In addition, schizophrenia participants with high social cognitive abilities still perform worse than healthy controls on measures of emotional awareness (18), suggesting that difficulties with emotional awareness in schizophrenia are not fully explained by overall social cognitive deficits. Despite strong evidence of reduced emotional awareness in schizophrenia-spectrum disorders, some large-scale studies have not observed impairments (7), indicating inconsistent findings. Therefore, identifying factors that relate to emotional awareness in schizophrenia is critical to better understanding how and when deficits may arise.

Emotional awareness is considered a relatively stable, trait-related construct in adulthood. Trait emotional awareness is influenced by emotional experience, which includes the generation, representation, and conscious access of affective responses [bodily sensations, cognitions, and motivated actions; for review, see (13, 19, 20)]. Bodily self-disturbances, such as impairment in interoceptive accuracy (i.e., the perception of one’s internal bodily signals) and anomalous emotional embodiment are observed in schizophrenia (21, 22). Altered bodily sensations and affective responses have been associated with positive symptoms of schizophrenia and psychotic-like experiences (23–25), suggesting that impairment in emotional awareness may also be linked with psychotic symptoms. Prior work has yielded mixed findings, with some evidence of more severe hallucinations and delusions related to lower attention to emotion (26) and other work finding no significant associations with positive symptoms after correcting for multiple comparisons exploring a multitude of symptom dimensions (17). *A-priori* examination of the hypothesis that lower emotional awareness is related to more severe positive symptoms of schizophrenia is therefore warranted.

Critically, emotional awareness develops throughout childhood, honed by social interactions (27, 28). Experiences with parents and caregivers support early learning and recognition of affective reactions (29) through experiences such as social referencing and emotional attunement between mother and child (30, 31). Emotion recognition is reduced in children raised in orphanages without personalized parental care, and consistently threatening environments can result in greater attention to external factors, limiting awareness of internal cues (32). Environmental factors, such as childhood adversity, have therefore been shown to disrupt development of emotional awareness, conferring risk for psychopathology (28, 33). In a longitudinal developmental cohort, low emotional awareness was found to mediate the relationship between exposure to childhood maltreatment and severity of psychopathology (p-factor) (34). Although childhood maltreatment is a general risk factor for psychopathology, it is consistently elevated in individuals with schizophrenia (35), suggesting that disruptions in emotional awareness during childhood may contribute to the signs or symptoms of schizophrenia.

Childhood maltreatment is also a risk factor for the experience of positive symptoms of psychosis, with work demonstrating elevated positive symptoms and psychotic-like experiences in those who have been exposed to abuse and victimization in childhood [for review, see (36)]. Interestingly, recent work has suggested a mediating role of emotion regulation on the relationship between maltreatment exposure and positive symptoms of psychosis in community-based samples (37, 38) and outpatients with a non-affective psychotic disorder (39). As noted above, emotional awareness is a critical precursor to emotion regulation, as awareness of one’s emotions and associated bodily sensations facilitates their regulation (40); in psychotherapy, raising emotional awareness is often a core skill of emotion regulation training (41, 42). Yet, to our knowledge, the relation between emotional awareness, childhood maltreatment, and positive symptom severity in schizophrenia has never before been investigated.

The current study aims to examine the association between emotional awareness, childhood maltreatment exposure, and positive symptom severity in individuals with schizophrenia. Specifically, it aims to accomplish the following: (1) confirm prior work demonstrating reduced emotional awareness in schizophrenia, using an objective measure (LEAS); (2) examine how emotional awareness relates to severity of positive symptoms in schizophrenia; (3) test the hypothesis that exposure to childhood maltreatment is related to worse emotional awareness (both continuously and categorically); and (4) explore whether low emotional awareness has an indirect effect on the relationship between self-reported maltreatment exposure and symptom severity in a cross-sectional sample.

If our hypotheses are supported, it would suggest that emotional awareness is an important target for the treatment of schizophrenia, as has been effectively shown in other populations [e.g., (43, 44)] that emotional awareness is impacted by early life experiences.

## 2 Methods

### 2.1 Participants

Forty-five (45) individuals with a schizophrenia-spectrum disorder (henceforth called schizophrenia participants) and forty-eight (48) healthy comparison participants with no history of psychiatric disorders were recruited (Table 1). Participants with schizophrenia were medically stable outpatients recruited from existing research databases managed by the Vanderbilt University Medical Center (VUMC) Psychotic Disorders Program. Healthy comparison participants were recruited through community advertisements. Diagnoses were confirmed by a Structured Clinical Interview of the DSM-IV-TR or DSM-V (SCID) (47) completed by a trained rater and signed off in a consensus meeting. Healthy control individuals are determined as not having any past or present psychological diagnoses or any first-degree relatives with a schizophrenia-spectrum disorder. Individuals with schizophrenia were included if they had a schizophrenia-spectrum diagnosis (19 schizophrenia, 8 schizoaffective, 16 schizophreniform, 1 psychotic disorder not otherwise specified). Of the 45 individuals with schizophrenia, 13 had a comorbid lifetime anxiety disorder and 18 had a comorbid lifetime depressive disorder. All participants were free of major physical or neurological illness, active substance use disorder, and significant head injury, and had an estimated premorbid IQ >75 according to the Wechsler Test of Adult Reading (45). One individual with a non-affective psychotic disorder was excluded for not completing the main task, resulting in 44 schizophrenia participants in the final analysis. Study protocol was approved by the Vanderbilt Internal Review Board (IRB) and all research participants signed a consent document prior to study participation.

**Table 1:** Demographics.

Demographics mean (standard deviation)	Healthy controls *N* = 48	Schizophrenia *N* = 44	Statistics: control vs. schizophrenia
Age	30.1 (7.8)	27.4 (5.6)	*t*(90) = 1.9, *p* = 0.15
Gender (male/female)	31/17	32/12	*χ*^2^ = 0.71, *p* = 0.4
Race (White/Black/Other)	33/10/5	28/13/3	*χ*^2^ = 0.1.13, *p* = 0.57
Premorbid IQ	114.3 (8.3)	105.1 (13.2)	*t*(90) = 4.01, *p* = 0.003
Personal education (years)	16.7 (2.3)	14.6 (2.4)	*t*(90) = 4.7, *p* < 0.001
Parental education (years)	14.7 (2.2)	14.7 (2.8)	*t*(90) = 0.07, *p* = 0.94
Depression	5.7 (6.3)	6.5 (7.8)	*t*(90) = 0.22, *p* = 0.64

Premorbid IQ measured with Wechsler Test of Adult Reading (45). Depression measured using the Beck Depression Inventory (46).

Participants were recruited between 2020 to 2021 and the majority of participants (92%) conducted the study at home on a personal laptop. Study engagement was monitored throughout the session by the experimenter through a zoom call with screen sharing. A smaller proportion of participants (8%) who did not have access to a laptop completed the study in-person.

### 2.2 Measures

#### 2.2.1 Levels of Emotional Awareness Scale

The Levels of Emotional Awareness Scale (LEAS) provides participants with short written descriptions of 10 different emotionally evocative scenarios, each followed by two open response questions asking participants how they (self) would feel and how the other person in the scenario (other) would feel (48) in the situation described. In this study, participants completed a 10-item Electronic Version of LEAS (eLEAS; http://eleastest.net/). The scenarios are designed to create a wide range of emotional reactions, including anger, fear, sadness, and happiness. The scenarios describe relatable but uncommon scenarios of everyday life which can range from benign to potentially life-threatening situations. The other person in the scenario ranges from complete strangers to loved ones which provides opportunities for participants to describe their feelings in a variety of situations. An example of a LEAS scenario is: “You and your best friend are in the same line of work. There is a prize given annually to the best performance of the year. The two of you work hard to win the prize. One night the winner is announced: your friend. How would you feel? How would your friend feel?” (49). The LEAS is a reliable and valid measure of individual differences in emotional awareness, and has previously been used in individuals with schizophrenia (9, 17).

The electronic version of the LEAS is unique in that it does not require hand scoring and is scored through a validated automatic scoring method (50). The Program for Open-Ended Scoring (POES) was developed as a scoring method for the eLEAS and has demonstrated good internal consistencies and validities that were comparable to hand scoring (51, 52). In this study, the POES was used to generate a score between 0 and 4 for self and other and 0 to 5 for total depending on the complexity of the response on each item (52). At the lowest score, a 0, the participant would write about non-emotional words or cognitions (e.g., I would be myself). A score of 1 includes the description of bodily sensations (e.g., I would feel pain), a score of 2 includes action tendencies (e.g., I would cry), and a score of 3 includes single emotions (e.g., I would feel angry). To score a 4 on self or other, the participant would write about two or more specific emotional words (e.g., I would feel happy and excited). To score a 5 for the total score, the participant would have to score a 4 in both self and other, showing a blend of emotional experiences for themselves and others with non-identical terms (e.g., I would feel sad and ashamed while the other person would feel happy and excited) (9, 48) [for specific examples see (49, 53)]. Scoring of the LEAS does not consider the congruity of emotions in the participants response to the scenarios. A total, self, and other score was calculated for each participant by summing the individually scored items, where higher scores indicate greater emotional awareness (54, 55).

The LEAS has been shown to report higher emotional awareness in female gender, older ages, and higher academic achievements (53). This is consistent with prior work on emotional awareness and deficits in emotional awareness (i.e., alexithymia) (56, 57). In order to address possible communication or mobility concerns, participants were allowed to complete the LEAS in an orally or written format which has been previously found to be insignificant to results (58).

#### 2.2.2 Positive and Negative Syndrome Scale

Psychiatric symptoms were measured in the schizophrenia group using the PANSS (59). The PANSS includes 30 items scored on a 7-point scale and assesses positive (7 items), negative (7 items) and general symptoms (16 items). The positive symptom items include delusions, disorganization, hallucinations, excitement, grandiosity, suspiciousness/persecution, and hostility. The PANSS is often used in clinical research and is considered a reliable, comprehensive assessment of psychotic symptoms (60). Cronbach’s alpha for the positive symptom scale was *α* = 0.73.

#### 2.2.3 Childhood Trauma Questionnaire-Short Form

The Childhood Trauma Questionnaire-Short Form (CTQ-SF) is a 28 item self-report measure that retrospectively assesses childhood emotional abuse and neglect, physical abuse and neglect, and sexual abuse before the age of 16 (61). Participants are asked to respond to questions, like “When I was growing up, people in my family hit me so hard that it left me with bruises or marks” and “When I was growing up, I did not have enough to eat.” Participants indicated how true these statements were to them, on a scale from never true (1) to very often true (5). The CTQ-SF has been shown to have a replicable factor structure, good validity with structured interviews, and convergent validity in both clinical and community samples (61, 62). The CTQ-SF also shows good sensitivity and convergent validity in both clinical and community sample (63).

Consistent with the literature, self-reported childhood maltreatment was considered both continuously and categorically for this study. Although some studies use CTQ scores continuously to assess the linear relationship between severity of self-reported maltreatment exposure and other phenomenon like depression (64), other studies have used CTQ scores to investigate group differences in those with self-reported, high, low and average exposure to childhood maltreatment (65) or categorical maltreatment and no maltreatment groups (66).

For categorical analyses, self-reported “maltreatment exposure” was based on the subscale cut-off scores defined in the CTQ manual (63). Subscale scores are measured as the sum of five items in each abuse subtype, falling into one of four groups: none to low self-reported maltreatment exposure, low to moderate self-reported maltreatment exposure, moderate to severe self-reported maltreatment exposure, and severe to extreme self-reported maltreatment exposure. “Maltreatment exposure” was defined as any CTQ subscale score endorsed as at least low to moderate maltreatment (Control *N* = 24, Psychosis *N* = 29). No maltreatment was defined as those who endorsed all CTQ subscale scores of none to low maltreatment exposure (Control *N* = 24, Psychosis *N* = 15). A similar proportion of healthy controls and schizophrenia participants were found in the maltreatment and no maltreatment exposure groups (*X*^2^ = 0.12, *p* = 0.14). Cronbach’s alpha our total sample is *α* = 0.77.

### 2.3 Statistical analysis

Analyses were performed in SPSS v.25.0 (67) and MatLab (68).

Shapiro–Wilk tests were first performed to assess the normality of each of the study variables. Given violations of normality in the distribution of PANSS Positive, Negative, and General symptoms (all *p*’s < 0.05) and PANSS positive symptom items (all *p*’s < 0.001), CTQ Total (*W* = 0.87 *p* < 0.001) and subscales scores (all *p*’s < 0.05), as well as Emotional Awareness-Self scores (*W* = 0.96, *p* = 0.004), non-parametric tests were used. Group differences in emotional awareness (total, self, and other) and childhood maltreatment (total and subscales) were assessed using independent samples Kruskal–Wallis test. Relationships between emotional awareness, psychiatric symptoms, and childhood maltreatment were tested using partial Spearman’s rho correlations controlling for age and sex. Lastly, we assessed whether emotional awareness had an indirect effect on the association between self-reported childhood maltreatment and positive symptom severity. This indirect effect analysis was conducted using the Process macro in SPSS (model 4), which determines confidence intervals for significance based on 5,000 bootstrap estimates. *A-priori* associations between emotional awareness, positive symptoms, and childhood maltreatment were uncorrected for multiple comparison, however follow-up post-hoc associations were Bonferroni-corrected.

## 3 Results

### 3.1 Group differences in emotional awareness

**Figure 1:**
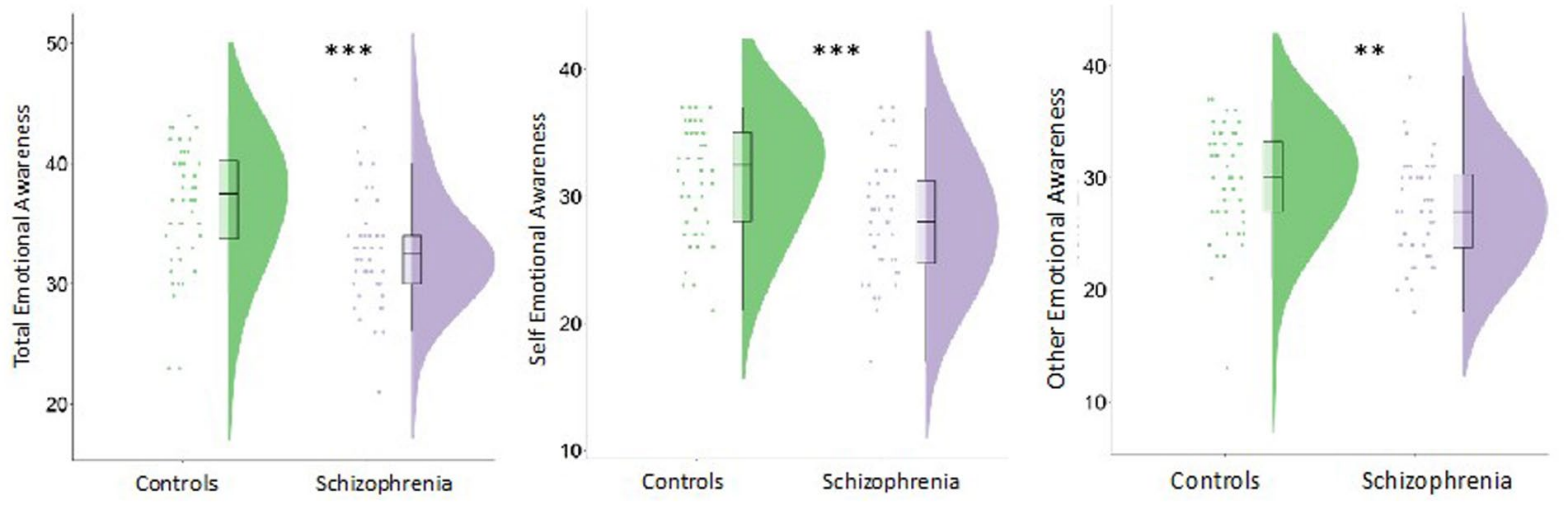
Individuals with schizophrenia demonstrated reduced emotional awareness (EA) overall (total), for the self, and for others ^***^p < 0.001 and ^**^p < 0.01.

Schizophrenia participants exhibited significantly impaired emotional awareness for total [*H*(1) = 13.1, *p* < 0.001, Cohen’s *d* = 0.76], self [*H*(1) = 12.0, *p* < 0.001, Cohen’s *d* = 0.78], and other [*H*(1) = 10.0, *p* = 0.002, Cohen’s *d* = 0.62] (Figure 1).

### 3.2 Relationships between emotional awareness and positive symptoms

**Figure 2:**
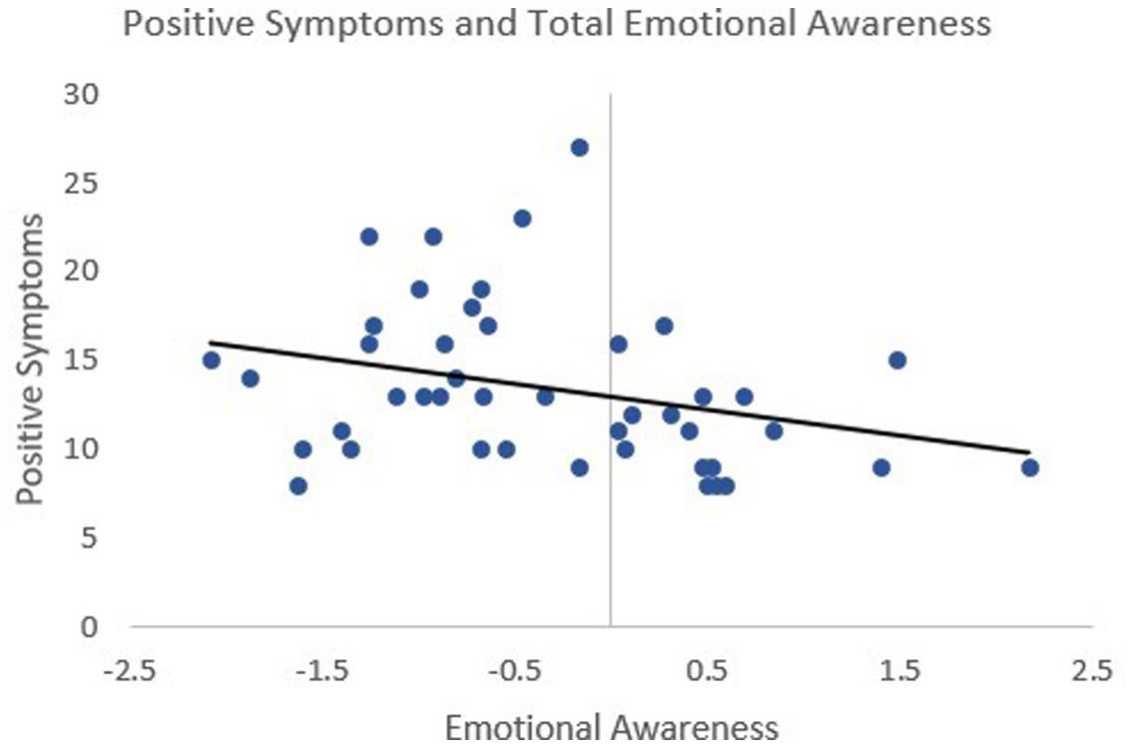
Higher positive symptoms were significantly associated with lower emotional awareness in schizophrenia (*p* = 0.42, *p* = 0.006) Emotional awareness was measured by the Levels of Emotional Awareness Scale (LEAS) and positive symptoms were measured through the Positive and Negative Syndrome Scale (PANSS). Emotional awareness scores are presented as residuals, controlling for age and sex, to reflect the analysis performed.

Greater impairment in total emotional awareness was significantly related to more severe positive symptoms in schizophrenia (*ρ* = −0.42, *p* = 0.006) (Figure 2). This association was observed for both emotional awareness of the self (*ρ* = −0.37, *p* = 0.017) and others (*ρ* = −0.37, *p* = 0.014).

Exploratory analyses were conducted to examine specificity with positive versus negative symptoms, and also to explore what aspects of the positive symptom scale were most strongly associated with overall emotional awareness. Total emotional awareness was not significantly related to negative symptoms (*ρ* = 0.07, *p*_corrected_ = 1). Of the positive symptoms, no individual symptoms were significantly associated with emotional awareness after correcting for multiple comparisons: disorganization (*ρ* = −0.36, *p*_corrected_ = 0.14), unusual thought content (*ρ* = −0.16, *p*_corrected_ = 1), hallucinations (*ρ* = −0.20, *p*_corrected_ = 1), suspiciousness (*ρ* = −0.24, *p*_corrected_ = 1).

### 3.3 Childhood maltreatment and emotional awareness

**Figure 3:**
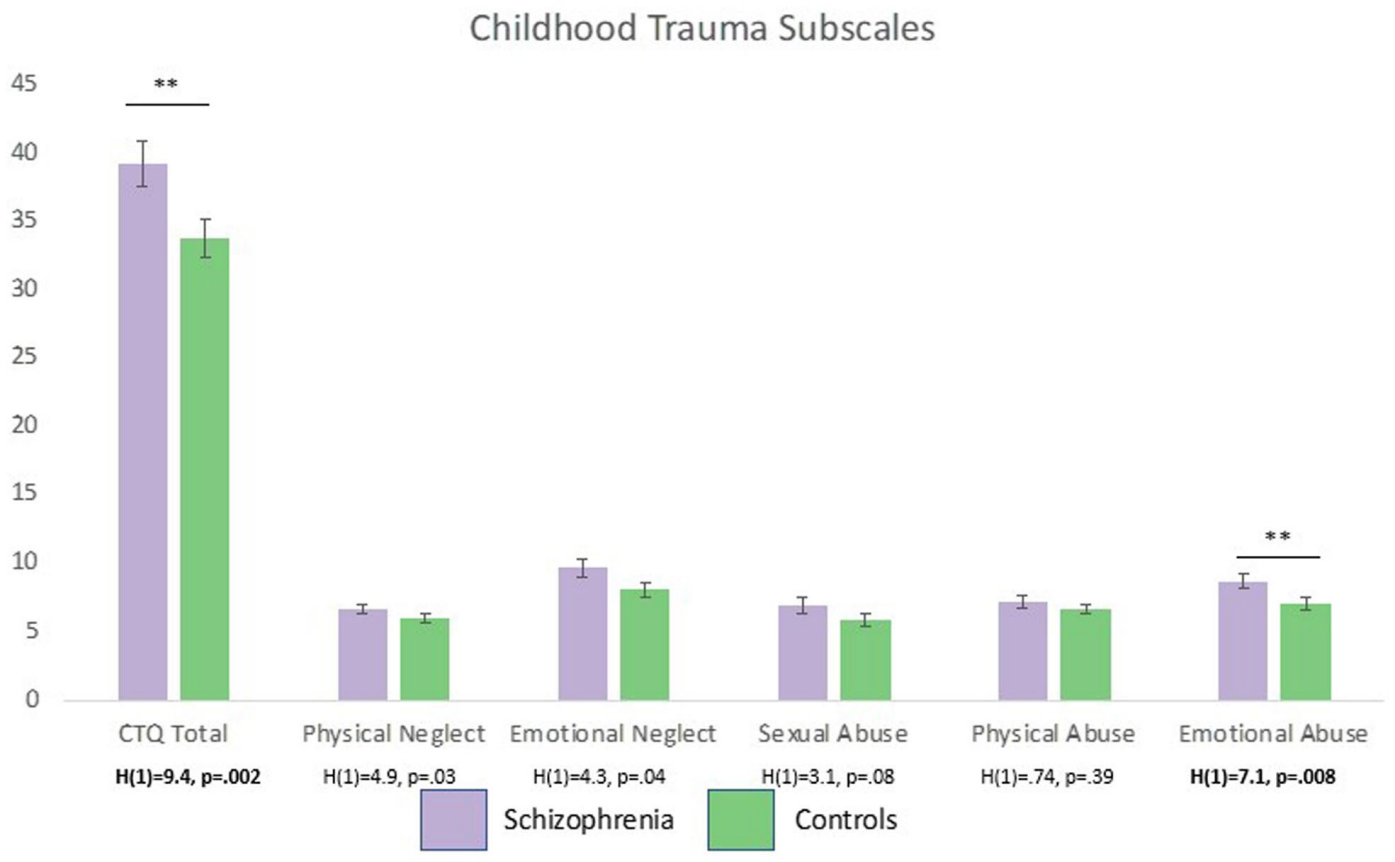
Individuals with schizophrenia self-reported significantly more severe childhood maltreatment than healthy controls. After controlling for multiple comparisons (critical alpha-01), only emotional abuse was significant elevated in patients compared to controls. ^**^p < 0.01.

Schizophrenia participants experienced significantly more severe childhood maltreatment than healthy comparisons participants [*H*(1) = 9.4, *p* = 0.002, Cohen’s *d* = 0.52]. After correcting for multiple comparisons, emotional abuse was the only sub-scale that was significantly elevated in participants with schizophrenia [*H*(1) = 7.1, *p*_corrected_ = 0.03, Cohen’s *d* = 0.49] (Figure 3).

**Figure 4:**
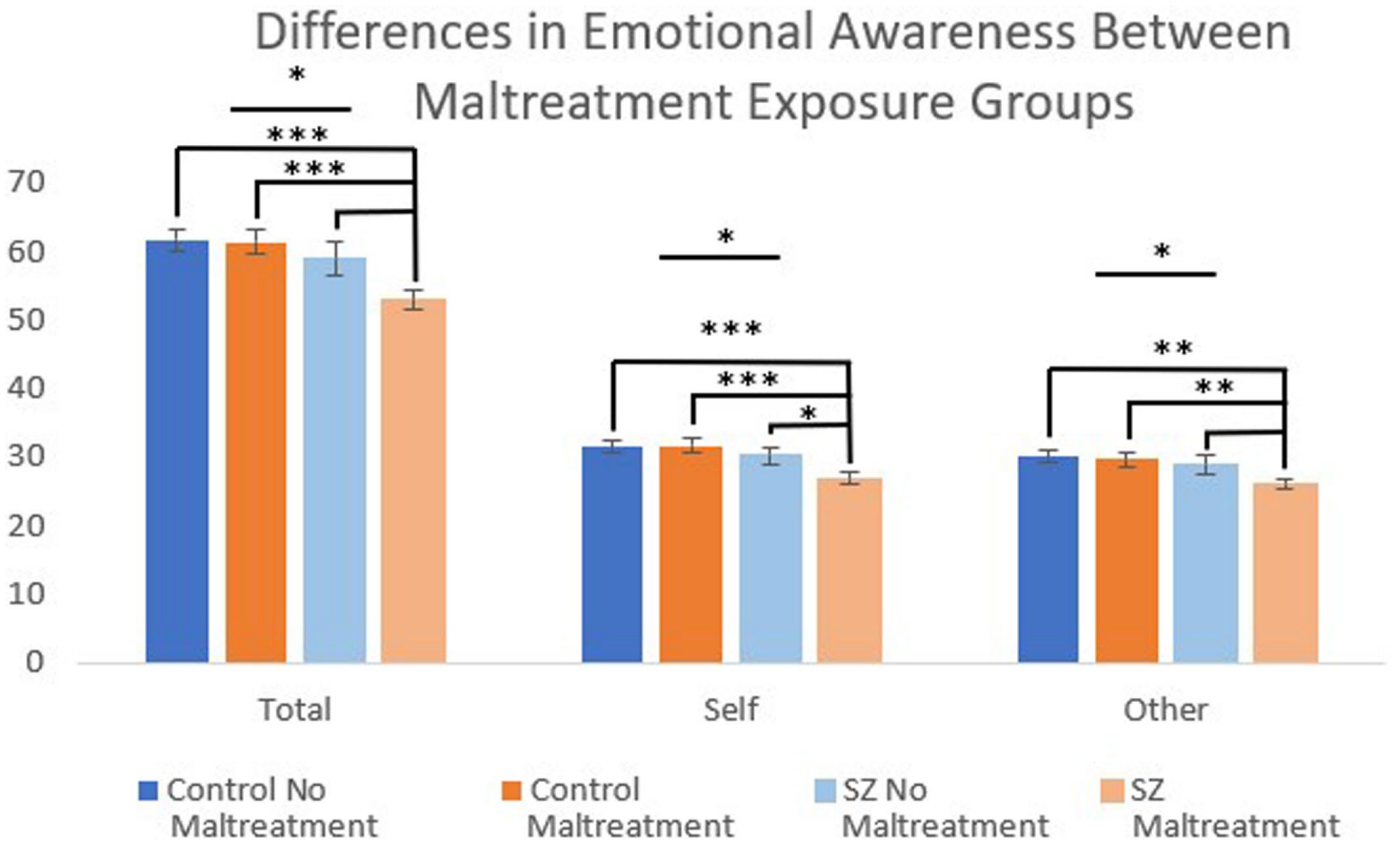
Individuals with schizophrenia exposed to childhood maltreatment (defined as at least one subscale rated as more than minimal) had significantly reduced emotional awareness compared with individuals without childhood maltreatment exposure. This included reduced emotional awareness of the self compared to psychosis without maltreatment. ^***^p < 0.001, ^**^p < 0.01, and ^*^p < 0.05.

When considering self-reported maltreatment exposure categorically, we found that the four groups (i.e., HC^+^, HC^−^, PSY^+^, PSY^−^) significantly differed on LEAS total [*H*(3) = 16.9, *p* < 0.001], self [*H*(3) = 16.5, *p* < 0.001], and other [*H*(3) = 12.9, *p* = 0.005] (Figure 4). This appeared to be driven by the psychosis group with maltreatment, which had lower LEAS scores than all three groups for total LEAS (PSY^+^ vs. PSY^−^: *p* = 0.05, PSY^+^ vs. HC^+^: *p* < 0.001, PSY^−^ vs. HC^−^: *p* < 0.001), self (PSY^+^ vs. PSY^−^: *p* = 0.04, PSY^+^ vs. HC^+^: *p* < 0.001, PSY^+^ vs. HC^−^: *p* < 0.001), and other (PSY^+^ vs. PSY^−^: *p* = 0.09, PSY^+^ vs. HC^+^: *p* = 0.002, PSY^+^ vs. HC^−^: *p* = 0.002) (all Cohen’s *d*’s > 0.57).

When looking continuously, overall childhood maltreatment severity was not significantly related to LEAS ability in schizophrenia (*ρ* = −0.17, *p* = 0.28) or across the whole sample (*ρ* = −0.17, *p* = 0.11).

### 3.4 Positive symptoms and childhood maltreatment

Positive symptoms in schizophrenia were not significantly elevated in those with exposure to childhood maltreatment [*H*(1) = 3.0, *p* = 0.08] and was not significantly related to severity of overall childhood maltreatment (*ρ* = 0.22, *p* = 0.16).

### 3.5 Indirect effect model of childhood maltreatment, emotional awareness, and positive symptoms in schizophrenia

Although positive symptoms were not significantly associated with self-reported childhood maltreatment in this sample, we tested our indirect effect model to examine whether low emotional awareness had an indirect effect on the association between self-reported childhood maltreatment exposure and positive symptom severity. In this sample, we did not find evidence of a significant indirect effect (95% CI, −0.04, 2.32, *p* > 0.05).

### 3.6 Exploration of impact of personal education and symptoms on emotional awareness scores

Given that LEAS scoring is based on language output, we conducted several analyses to examine whether education and/or specific symptoms were accounting for individual differences in scores. We found that, when personal education was included as a covariate, individuals with schizophrenia continued to have significantly lower emotional awareness (all *p*’s < 0.02). In addition, total emotional awareness was not significantly associated with stereotyped thinking, lack of spontaneity and flow of conversation, difficulty with abstract thinking, or flat affect on the PANSS (all *p*’s > 0.14).

## 4 Discussion

This study is one of the first to investigate associations between emotional awareness, childhood maltreatment exposure, and positive symptom severity in schizophrenia. Our findings add to an existing literature on emotional deficits in schizophrenia by demonstrating impaired emotional awareness (for both self and others) in patients with schizophrenia using a performance-based measure. We also show that lower emotional awareness is associated with more severe positive symptoms, suggesting that poorer identification and interpretation of emotions may contribute to “reality distortions,” such as delusions and hallucinations. In addition, although childhood maltreatment was not continuously related to emotional awareness, patients with schizophrenia who reported exposure to childhood maltreatment had worse emotional awareness than healthy controls and schizophrenia participants without self-reported exposure to childhood maltreatment. Notably, in this sample, we did not find evidence of a continuous association between severity of childhood maltreatment and positive symptom severity, and we did not find evidence that emotional awareness had an indirect effect on the association between self-reported childhood maltreatment exposure and positive symptoms. Future studies in larger samples may be better able to detect these associations if they exist. Taken together, our findings suggest that emotional awareness deficits in schizophrenia relate to severity of positive symptoms, and that emotional awareness may be most impacted in those who have experienced childhood maltreatment.

A large body of work has investigated disturbances in emotional processes in individuals with schizophrenia, finding that schizophrenia is associated with impairment in emotional expression, emotion recognition, and emotion regulation (1, 2, 6, 69, 70). Alexithymia is also an impairment in schizophrenia, capturing a general deficit in the ability to identify, describe, and communicate feelings (5). Emotional awareness is a facet of alexithymia that focuses specifically on the ability to identify and put into words the feelings of oneself and others, but it does not extend to deficits in imaginal ability or externally-oriented thinking as is measured with alexithymia (8, 55, 71, 72), and it is distinct from the construct of alexithymia (72). While deficits in emotional processes are commonly reported in schizophrenia, there have been inconsistencies in the findings on emotional awareness (26, 72). One recent report using the LEAS failed to identify emotional awareness alterations for total, self, or other in individuals with schizophrenia (17). Other studies have reported lower LEAS scores in schizophrenia for the self, but not other or total scores (15), as well as no differences in the LEAS for those with schizotypy (7). Therefore, although emotional awareness deficits are often observed in schizophrenia, as they were in the current study, identifying factors that may contribute to altered emotional awareness, and that can explain some of these discrepancies, is critical for accurate conceptualization of how these deficits arise.

In this dataset, emotional awareness was lower in patients with schizophrenia who self-reported childhood maltreatment exposure as compared with patients who did not, most strongly for emotional awareness of the self. This finding may explain some of the above-noted mixed findings of altered emotional awareness in schizophrenia and suggests that early childhood adversity influences how individuals experience and understand affective responses later in life. Smith et al. (13) theorized multiple mechanisms that could impact individual differences in emotional awareness, including early learning of emotions during childhood. Additionally, early maltreatment can affect a person’s neurobiological mechanisms which may then influence the development of psychotic-like experiences and deficits in affective abilities later in life (73–75). For instance, active inference is a neurocomputational mechanism thought to underlie difficulties with emotional awareness (27) that has also been linked to childhood maltreatment (76). Under predictive coding models, childhood maltreatment may impact learning from the environment, which would have downstream effects on expectations about the emotions of oneself and others. Social interactions scaffold the development of emotional functioning throughout development, through processes like social referencing (77–79). Conversely, neglect has been linked to difficulties with emotion recognition, awareness and regulation (31, 80–82).

Childhood maltreatment is also a strong risk factor for schizophrenia (83). In the current study, self-reported childhood maltreatment was elevated in the schizophrenia participants, although not all individuals with schizophrenia endorsed this exposure. Some authors proposed a “subtype” of schizophrenia characterized by trauma-related dissociative experiences (84). This subtype includes patients with schizophrenia who have increased dissociative experiences, childhood maltreatment exposure, and extrasensory perceptions. Recent work points to the mediating role of self-disturbances in the relationship between maltreatment and psychotic-like experiences (85–87). While the current study did not identify a significant association between childhood maltreatment and positive symptoms, data on dissociation may have helped clarify this link, which represents an important avenue for future investigation (88).

Positive symptoms were, however, significantly related to emotional awareness, such that more intense positive symptoms were associated with worse performance on the LEAS. This was true for the total LEAS, self, and other scores. In schizophrenia, affective deficits have largely been studied in the context of negative symptoms like blunted affect and social anhedonia (14, 89, 90). However, there is a growing body of literature that has found connections between positive symptoms and emotional deficits (26, 91–93), including emotional awareness (94, 95). Emotional awareness relies on interoception, which is disrupted in schizophrenia (21, 96) and has been associated with positive symptoms (24). Notably, in the current sample, positive, but not negative, symptoms were associated with emotional awareness; yet, within the positive symptom domain, there was little specificity, as it was only positive symptom severity overall that showed significant correlations. While more research is needed to understand how and why emotional awareness relates to positive symptoms, it is clear that accurate emotional awareness allows us to adaptively interpret the social world. Inaccuracies or blunting in the ability to notice, label, and interpret emotions can distort one’s current reality and impair interoceptive predictive coding processes that generate cohesive meaning about our experiences and have been suggested to account for dissociative experiences in a variety of psychiatric disorders (27, 96–99).

These findings present important clinical implications for patients with schizophrenia. Findings suggest the importance of taking a full social history of patients, including childhood maltreatment exposure, in order to begin assessing for and conceptualizing the nature of emotional difficulties in the patient, including emotional awareness. In addition, therapies specifically targeting the improvement of awareness may be particularly useful in these patients and should be considered. This is especially true if more complex emotional processes, such as emotion regulation and/or positive symptoms are to be worked on. Metacognitive Reflection and Insight Therapy [MERIT (100)] is one example of this type of therapy, developed for individuals with schizophrenia (101). This meta-cognitive based therapy has been found to show continued improvement of meta-cognitive abilities and emotion regulation in follow up (102, 103), possibly facilitating or being facilitated by emotional awareness skills. Furthermore, the use of objective measurement of emotional awareness such as the eLEAS provides researchers with a tool to gauge patient’s abilities and change over time in clinical and non-clinical settings, which could be used to assess improvement with treatment.

This study was limited by a relatively small sample size, which may have affected the power to detect each hypothesized relationship and, more importantly, the proposed indirect effects model. Consistent with past research, [*n* = 631, rho = 0.15–0.32 (104);], this study found weak strength between positive symptoms of psychosis and childhood maltreatment. Post-hoc power analysis revealed we had only 47% power to detect a significant indirect effect with our current effect sizes. Furthermore, as an indirect effects model, this data does not allow for causal conclusions and therefore future directions may address this. Another limitation is the retrospective nature of the Childhood Trauma Questionnaire, which may have impacted the reliability of the maltreatment reports. The CTQ is also not well suited to assess other childhood stressors (e.g., neighborhood violence) that might also impact emotional functioning. Additionally, the majority of participants completed the study remotely, providing less control of the study environment. Lastly, the data did not include neuroimaging procedures which could have further supported group differences in emotional awareness.

### 4.1 Future directions

These data are some of the first to directly examine relationships between emotional awareness, childhood maltreatment, and symptomology in schizophrenia, and they support continued exploration of these associations in independent samples. If replicated, future studies should examine the timing of the relationships studied here; for example, examining whether these relationships can be observed with prodromal symptoms of schizophrenia and at which age maltreatment occurred. In addition, while we hypothesize that childhood maltreatment contributes to lower emotional awareness, which in turn contributes to more severe positive symptoms, the ability to test this hypothesis is limited in a cross-sectional sample. Longitudinal assessment of these factors would provide better understanding of how they relate over time, and whether they contribute to perpetuating patterns of emotion dysregulation and distress that can contribute to psychotic experiences (38). Emotional granularity, individual differences in one’s ability to distinguish their emotional states (105), represents a way to establish differences in affective abilities using correlations among self-reported emotions longitudinally. While emotional awareness has been found to have group differences in sex, age, and education level, emotional granularity represents an additional avenue to examine abilities with less risk to validity due to group differences in demographic factors. Finally, this study provides support for considering emotional awareness as an important factor in symptom severity, and as a potential target for treatment in patients with schizophrenia, as has been previously suggested (106, 107). This highlights the importance of studying individual differences, like childhood maltreatment, in symptom presentation and treatment.

## Data availability statement

The raw data supporting the conclusions of this article will and be made available by the authors, without undue reservation.

## Ethics statement

The studies involving humans were approved by Vanderbilt University Medical Center Institutional Review Board (IRB #: 201489). The studies were conducted in accordance with the local legislation and institutional requirements. The participants provided their written informed consent to participate in this study.

## Author contributions

KB: Conceptualization, Data curation, Formal analysis, Project administration, Resources, Visualization, Writing – original draft, Writing – review & editing. LT: Formal analysis, Resources, Validation, Writing – review & editing. RS: Resources, Validation, Writing – review & editing. RL: Resources, Software, Validation, Writing – review & editing. JS: Conceptualization, Data curation, Formal analysis, Funding acquisition, Investigation, Methodology, Project administration, Resources, Supervision, Validation, Writing – original draft, Writing – review & editing.
